# Abilities of herbaceous plant species to phytoextract Cd, Pb, and Zn from arable soils after poly-metallic mining and smelting

**DOI:** 10.1007/s11356-025-36241-6

**Published:** 2025-03-17

**Authors:** Michael O. Asare, Elisa Pellegrini, Jiřina Száková, Jana Najmanová, Pavel Tlustoš, Marco Contin

**Affiliations:** 1 Department of Agroenvironmental Chemistry and Plant Nutrition, Faculty of Agrobiology, Food, and Natural Resources, Czech University of Life Sciences Prague, Kamýcká 129, 165 00 Prague 6, Czechia; 2 Department of Agricultural, Food, Environmental, and Animal Sciences, University of Udine, Via Delle Scienze 206, 33100 Udine, Italy

**Keywords:** Alluvial sediment, *Equisetum arvense*, *Leucanthemum vulgare*, Phytoextraction, Phytoremediation, Shoot accumulation

## Abstract

Potentially toxic element (PTE) contamination deteriorates agricultural land. This study explored the accumulation of excess PTEs (Cd, Pb, and Zn) in soils by shoots of herbaceous plants growing on alluvial sediments of an abandoned mining/smelting site near the Litavka River, Czech Republic, as a means of soil remediation. Determination of total Cd, Pb, and Zn, contents in soil and plant samples decomposed with HNO_3_ + HCl + HF, HNO_3_, and H_2_O_2_, respectively, were carried out by inductively coupled optical emission spectrometry. The soil Cd, Pb, and Zn contents in the studied site ranged from 40 to 65, 3183 to 3897, and 5108 to 6553 mg kg^−1^, respectively, indicating serious soil contamination compared to the limits allowed by the FAO/WHO and the Czech Republic. Slightly acidic soil reactions and negative correlations between the pH, C, and N supported the assumption of relative solubility, mobility, and accumulation of studied PTEs by herbaceous species. Shoot accumulation of Cd, Pb, and Zn varied in 22 of 23 species recording a Cd content above the permissible limit. The Zn content in all plants was above the WHO limit. Except for *Arabidopsis halleri*, with a bioaccumulation factor (BAF_shoot_) > 1 for Cd and Zn, *Equisetum arvense* recorded a comparatively higher Cd content (10.3–28 mg kg^−1^) than all other species. *Silene vulgaris* (Moench), *Leucanthemum vulgare*, *E*. *arvense*, *Achillea millefolium*, *Carex* sp., *Dianthus deltoides*, *Campanula patula*, *Plantago lanceolata*, and *Rumex acetosa* accumulated more Zn than many plants (> 300 mg kg^−1^). Although *E*. *arvense* had a BAF < 1, it accumulated > 1000 mg Zn kg^−1^ and supported the phytoextraction of Zn. Only 10 species accumulated Pb above the limit permissible in plants, with *L*. *vulgare* recording the highest concentration (40 mg kg^−1^) among all species. Therefore, the shoots of several plant species showed promising PTE accumulation abilities and deserve more detailed studies concerning their potential use for phytoremediation of Cd-, Pb-, or Zn-contaminated soils.

**Supplementary Information:**

The online version contains supplementary material available at 10.1007/s11356-025-36241-6.

## Introduction

Excessive accumulation of potentially toxic elements (PTEs), e.g. cadmium (Cd), lead (Pb), and zinc (Zn), in soils can be detrimental to plants (Okereafor et al. 2020; Alengebawy et al. 2021; Yang et al. 2023). However, although Zn is a micronutrient supporting plant physiological and metabolic functions, it can also become phytotoxic when in excess (Pavlović et al. 2021). However, Cd and Pb have no known relevance to soil biota or plant growth and are phytotoxic upon exceeding certain species-dependent thresholds. Knowledge of plants with a high accumulation of PTEs is a prerequisite for species suitable for cleaning PTE-contaminated soils. Various human activities can lead to rapid accumulation of PTEs in the soil. Abandoned mining sites near a Pb/Zn smelter in southwestern China retain high ecological loads of Cd (75), Pb (2485), and Zn (8078 mg kg^−1^) (Li et al. 2015), which are significantly above the limits permissible in agricultural soils: 0.5 Cd kg^−1^, 60 mg Pb kg^−1^, and 120 Zn mg kg^−1^ (WHO 1996). PTE-contaminated soils are unsuitable for food crops and forage (Bernal et al. 2020) and require extensive remediation. Such conditions make the management of polluted sites often unsustainable.

Studies have shown that several plants can efficiently accumulate Cd, Pb, or Zn in tissues and organs thus suitable for cleaning contaminated soils, i.e. phytoremediation (Berhongaray et al. 2015). For example, *Fragaria vesca* and *Potentilla arenaria* show high root accumulation of Cd, Pb, Zn, and other toxic elements (Stefanowicz et al. 2016)*.* However, root accumulation can result in a threat due to the high possibility of the re-accumulation of the PTE in the soil, especially during root exudation (Chen et al. 2017*)*. In this context, the substantial removal of risk elements by plants has been reported for constructed wetlands, where emergent macrophytes, such as *Phragmites australis*, seemed to be effective for the removal of risk elements from mine wastewater (Githuku et al. 2021). These authors reported a Cd and Pb removal efficiency exceeding 90% and ≈ 75%, respectively. *Phragmites australis* plants growing in mine tailings ponds, however, were able to accumulate risk elements, including Zn, Pb, and Cd, in roots, indicating their potential for phytostabilisation of the polluted area (Prica et al. 2019).

In the terrestrial environment, Luo et al. (2022) reported the effective phytostabilisation of the area polluted by a smelting waste slag using *Lolium perenne* and *Trifolium repens*. In the case of polluted soils or sediments, the phytoextraction efficiency depends not only on the ability of plants to accumulate these elements but predominantly on soil properties and risk element mobility in these soils (Prica et al. 2019). The role of soil physicochemical parameters and plant properties in Pb accumulation in various plant species was recently reviewed by Kumbhakar et al. (2023). Soil chemical properties, such as soil reaction (pH) and element content, together with plant exudates govern the mobility of PTEs and contribute to their accessibility (Hazelton and Murphy 2011; Sungur et al. 2021). These parameters individually or collectively play a vital role in the accumulation abilities of different herbaceous plants in a given location.

Species including *Noccaea caerulescens* (Mohtadi et al. 2012), *Silene vulgaris* Moench (Pradas del Real et al. 2014), *Biscutella laevigata* (Pošćić et al. 2015), and *Agrostis capillaris* (Teodoro et al. 2020) have exhibited high tolerance to Zn, Pb, and Cd. In particular, PTE accumulation in shoots/aerial organs has been considered a much safer mode of phytoremediation (Siyar et al. 2022) than the roots of plants, e.g. perennial plants (Zárubová et al. 2015; Asare et al. 2024). Grasses, such as moor grass, accumulate a significant Zn, Cd, and Pb content in soils heavily contaminated with Zn and Pb from ore-tailing landfills (Pietrzykowski et al. 2018). *Taraxacum* sect. *Ruderalia* and *Polygonum aviculare* accumulate up to 1.9 and 2.9 kg of Cd mg^−1^, respectively, presenting a substantial health hazard to *Oryctolagus cuniculus* living within the area (Hanousková et al. 2021). Compared to conventional methodologies for remediating contaminated fields, e.g. excavation and washing, aboveground accumulation of PTE by plants has other benefits, such as soil nutrient replenishment (Suman et al. 2018) and the re-emergence of shoots, especially perennial plants, after roots remain un-uprooted, potentially resulting in re-accumulation (Verma et al. 2021; Alsafran et al. 2022).

Plant species of the Pteridaceae (e.g. *Pteris vittata*) and Brassicaceae (e.g. *Noccaea caerulescens*) families show high consistency in hyperaccumulation (aerial organ of plant accumulation) of excess arsenic (As), Cd, and Zn (Małecka et al. 2019; Castaňares and Lojka 2020). Additionally, *Viola baoshanensis* and *Silene gracilicanlis* recorded shoot accumulation of 19.710 Zn mg kg^−1^ and 3.617 Pb mg kg^−1^, respectively (Wang et al. 2008). Additionally, ferns such as *P*. *vittata* are known for their hyperaccumulation of several 1000 mg kg^−1^ of As, while aquatic ferns (*Salvinia minima*, water moss) are also known for the aerial accumulation of excess Pb (Estrella-Gómez et al. 2009; Asare et al. 2023b, a).

Plant species spontaneously growing in mine tailings often demonstrate good bioaccumulation for the risk elements. For instance, *Vachellia campechiana* (Fabaceae), *Gliricidia sepium* (Fabaceae), and *Dodonaea viscosa* (Sapindaceae) growing at the mine tailings in specific areas in Mexico have been demonstrated as promising candidates for phytoremediation (either phytoextraction or phytostabilisation) of Cr, Cu, Cd, Zn, Pb, and other risk and essential elements (Santoyo-Martínez et al. 2020; Castañeda-Espinoza et al. 2023; Mussali‑Galante et al. 2023). Field surveys of abandoned metallurgical and extensive agricultural sites are often occupied by PTE-tolerant herbaceous species (Musilova et al. 2016) that accumulate excess PTEs in their organs (Ricachenevsky et al. 2021; Asare et al. 2024). Yang et al. (2022) evaluated the potential risk of toxic elements accumulation in plants growing in a grassland system in a mining area and the impact of soil and plant pollution on human and animal health. They analysed plants representing the Poaceae, Amaranthaceae, Nitrariaceae, and Tamaricaceae families, with various risk assessment indices indicating potential environmental hazards of the soil and plant pollution levels. Similarly, Sardar et al. (2021) reported that medicinal plants growing in degraded mining soils were characterised by high levels of risk elements, including Cd, Pb, and Zn. Thus, they can assist in the phytoextraction of PTEs (metal removal) from contaminated sites, which is a permanent approach to cleaning metal- or metalloid-contaminated soils (Moreira et al. 2021). PTE-accumulating species can be used for phytoremediation (removal of contaminants from soils) or phytomining (growing plants to harvest metals). However, herbaceous plants are fast-growing, self-sustaining, and predominantly perennial. Thus, persistent herbaceous plants for PTE phytoremediation can offer a cost-effective, time-saving, and lasting approach suitable for contaminated sites (Gajić et al. 2018). Due to the health benefits of many herbaceous species (e.g. secondary metabolites and their derivatives), considerable attention is required when analysing PTEs, especially in species growing at contaminated sites (Asare et al. 2022).

Příbram represents a hotspot of soil contamination in the Czech Republic, predominantly from previous mining and smelting of Pb, Ag, and Zn polymetallic ores (Ettler et al. 2005; Vaněk et al. 2008). Geochemical data (Borůvka and Vácha 2006) and magnetic susceptibility methods (Dlouhá et al. 2013) have revealed high anthropogenic sources of Cd, Pb, and Zn in soils. Surveying and analysing spontaneous plants that grow in contaminated sites is an efficient way to identify species suitable for PTE accumulation (Chapman et al. 2019) and to provide reliable inferences on possible bioaccumulation in the food chain. Thus, the study explored herbaceous plants persistently found in Cd-, Pb-, and Zn-contaminated soils in Příbram under field conditions to support the management of contaminated sites for arable land. The enrichment factors of PTEs in shoots of herbaceous plants are well estimated from threshold limits, e.g. by the World Health Organization (WHO) and other regulatory values. Moreover, due to evolutionary diversity, plants can exhibit variations in their PTE accumulation abilities. The current study hypothesised that herbaceous species that grow in PTE-contaminated soils can be used to recover such fields even for agricultural purposes due to their susceptibility to contaminated sites, as preference is given to species with high shoot accumulation.

This study, therefore, aimed to assess herbaceous plants with a broad spectrum of regional and global distribution and their ability to accumulate Cd, Pb, and Zn in their shoots from contaminated sites under different soil chemical properties, including pH, cation exchange capacity (CEC), and carbon (C) and nitrogen (N) contents. Such studies contribute to the identification and precise selection of more suitable herbaceous plants that might offer an economically friendly approach to decontaminating contaminated soils or assisting major phytoremediation schemes. The results of this study can also help to evaluate PTE-contaminated sites for arable field purposes.

## Materials and methods

### Study area

**Fig. 1:**
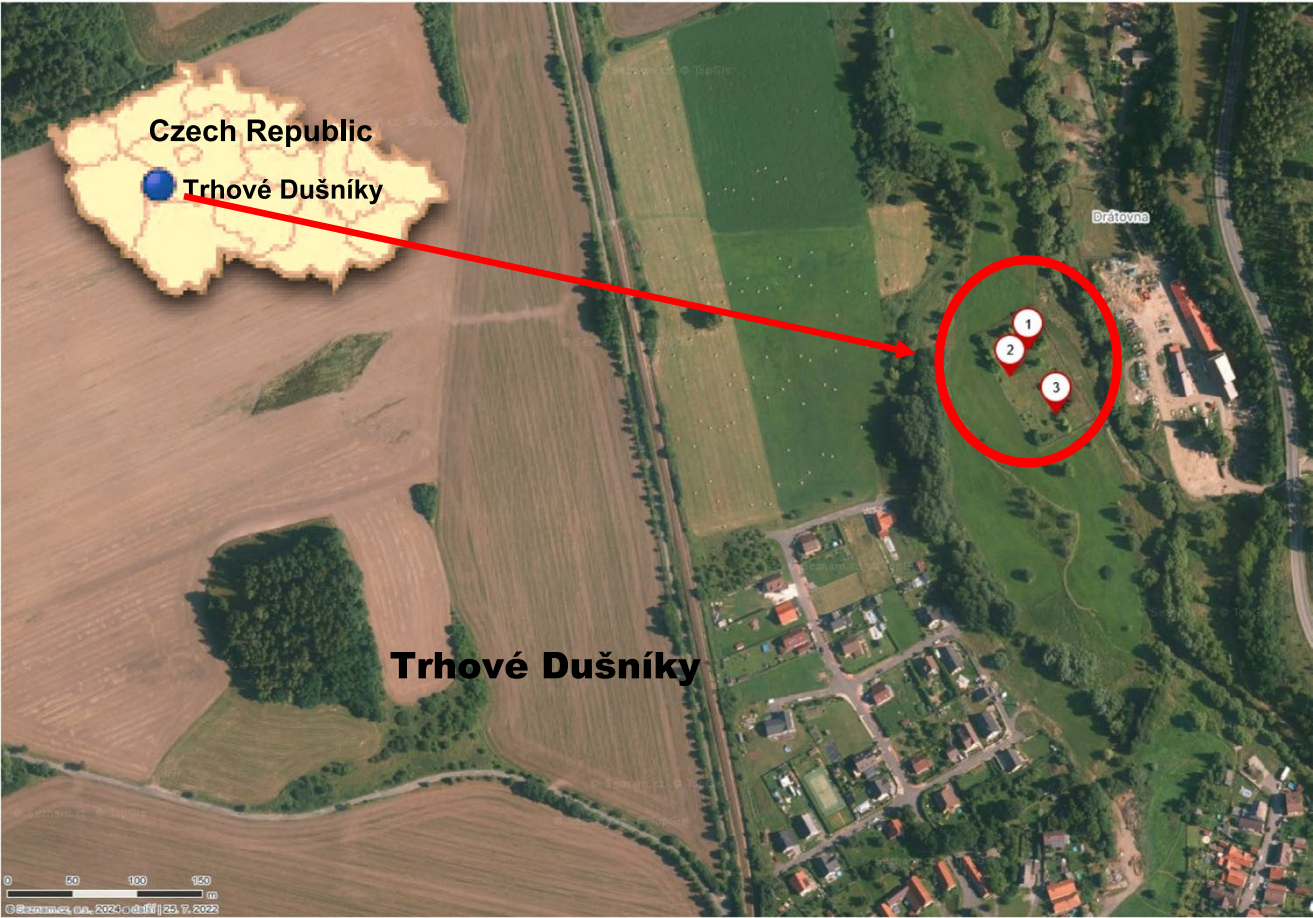
The map showing the location of the studied contaminated site in Trhové Dušníky village, Czech Republic; LKA (1), LKB (2), LKC (3)

The study site was located at Trhové Dušníky (49° 42′ 59.99″ N, 14° 00′ 60.00″ E) on approximately 1 ha forming part of the catchment of the Litavka River (Dlouhá et al. 2013) within the Příbram District a. 69 km SW of Prague, Czech Republic (Fig. 1). The site is 450 m above sea level with an average annual air temperature and precipitation of 7.5 °C and 600–800 mm, respectively.

The soils in the area result from sediments from the flood plain (fluvial/alluvium) of the Litavka River (Faměra et al. 2018; Kotková et al. 2019) located on schists, sandstones, grey wacks, and quartz bedrocks (Czech Geological Survey 2012). The site characterises other minerals, e.g. galenite (PbS), sphalerite (ZnS), boulangerite (Pb_5_Sb_4_S_11_), and antimonite (Sb_2_S_3_) rich in multiple PTEs (Borůvka and Vácha 2006).

### Soil sampling and analysis

The selection was based on three open fields with clusters of different herbaceous species with approximately 35 m × 35 m for each part, namely LKA, LKB, and LKC of the entire studied site. To cover the variability of soil samples and prevent overlapping, ix soil samples were randomly collected at each location, LKA, LKB, and LKC, where herbaceous plants were distributed. This sample collection was used because plants generally directly influence the soil’s chemical properties.

Each sample represented a composite sample of 6 sub-samples from the sampling square (5 m^2^) at a 0–30 cm depth. In total, 9 soil samples were collected. Soil samples were air-dried for 72 h, homogenized through a 0.2-mm sieve, and the total and mobile contents of Cd, Pb, and Zn were determined.

The soil pH (CaCl_2_) was measured using a 1/2.5 (w/v) suspension of soil and 0.01 M calcium chloride (CaCl_2_). The CEC was also determined using a barium chloride solution (ISO 11260:^2018^ procedure). The total C and N contents were determined using a CNS elemental analyser Vario MACRO CNS cube (Elementar Analysensysteme GmbH, Langenselbold, Germany).

To determine the total element contents, the extraction mixture comprised 65% nitric acids (HNO_3_), 36% hydrochloric acids (HCl), and 38% hydrofluoric acids (HF), as described by Asare et al. (2024). Briefly, a homogenized soil sample of 0.25 g was mineralised in a mixture of 9 mL HNO_3_, 3 mL HCl, and 1 mL HF and heated in a sealed 60 mL VWR PTFE Jar on a hot plate at 150 °C for 24 h. After 24 h, 1 mL of 30% hydrogen peroxide (H_2_O_2_) was added to each sample and evaporated on a hot plate at 50 °C for 24 h. The evaporated samples were then diluted to 20 mL with 2% HNO_3_ and filtrated. The mobile proportions of the elements were determined in the 1 M NH_4_NO_3_ extracts (DIN ISO 19730 2008). Briefly, 2 g of homogenized soil was shaken with 20 mL of 1 M NH_4_NO_3_ solution in a horizontal shaker for 2 h, centrifuged at 3000 rpm, and filtered. The total and mobile contents of elements in the digests were determined with inductively coupled plasma optical emission spectrometry (ICP-OES, Agilent 720, Agilent Technologies Inc., USA).

### Sampling and preparation of herbaceous plants

The selection and geobotanical interpretation of herbaceous plants followed the identification of mature persistence species with a high presence (Misra 2013; Asare and Száková 2023). The nomenclature of plants according to taxa followed the Angiosperm Phylogeny Group-APG IV (https://www.gbif.org/species/3106). We collected all plant samples from June to August 2022, the vegetative period of the area.

Plant samples were randomly collected to cover the locations with the spontaneous growth of herbaceous species at each location. Two mature samples of the same herbaceous plant from each location were collected based on screening. *Crepis biennis*, *Deschampsia caespitosa*, *Rumex acetosa*, *Rumex obtusifolius*, and *Chaerophyllum aromaticum* grew above 1 m, and shoots were harvested 0.2 m above the belowground biomass in all sampled locations. The collected samples were sent to the laboratory in plastic bags.

The plant samples were well-cleaned of environmental pollutants using deionised water and an ultrasonic bath. The samples were oven-dried in an electric oven (SLW 53 STD, POL-EKO, Wodzisław Śląski, Poland) at 60 °C for 3 days and milled (steel Retsch friction, Haan, Germany, particle size 0–1 mm). Homogenized plant samples were analysed for the total Cd, Pb, and Zn contents in two replicates.

### Analytical methods and procedures for plant samples

An aliquot of ~ 500 mg dry matter from each homogenized plant sample was digested in 8.0 mL of concentrated HNO_3_ and 30% H_2_O_2_ (2.0 mL) obtained from Analytika Ltd. (Czech Republic). The mixture was heated in an Ethos 1 (MLS GmbH, Germany) microwave-assisted wet digestion system for 30 min at 220 °C. Furthermore, the mixture was cooled and conveyed into a 20-mL glass volumetric flask, topped with deionised H_2_O, and maintained at laboratory temperature. The digest was then determined for the total element content with ICP-OES (Agilent 720, Agilent Technologies Inc., USA) in triplicate (Asare et al. 2024).

Standard reference material (SRM; NIST SRM dried tomato leaves 1573; http://www.nist.gov/srm) certified for the determination of the contents of control elements (total Cd and Zn) in dry weight of plant biomass was analysed to test the reliability of the analytical method. The analysis of the SRM obtained values of 1.35 ± 0.1 for Cd and 28.6 ± 0.9 for Zn against the original SRM values of 1.52 ± 0.04 for Cd and 30.9 ± 0.7 for Zn. The control elements provided a percentage recovery (*R*) > 85 (Magnusson and Örnemark 2014), indicating adequate precision of the analytical procedure.

### PTE accumulation index

The average levels of toxic elements in plants are controlled by the contents of PTEs. PTE toxicity directly affects the physiology, structure, and growth of plants. The bioaccumulation factor (BAF _shoot_) was estimated according to Mesa et al. (2017).

$$BAF\text{shoot}=\frac{\text{content of PTE in shoot }(\text{dry weight})}{\text{content of PTE in soil }(\text{dry weight})}$$

*BAF*_shoot_ > 1 indicates enrichment of the plant structures by PTEs (accumulators).

### Determination of individual soil pollution index

The pollution index was estimated and interpreted according to Chen et al. (2015).

$${I}_{i} =\frac{{C}_{i}}{{S}_{i}}$$

where Ci is the content of the measured PTE, and Si is the permissible limit of the evaluated PTE. In this case, the preventive levels of elements in soil, as given by Czech Public Notice No. 153/2016 (Cd, 0.5; Pb, 60; Zn, 50 mg kg^−1^) are considered Si.

(i) Ii ≤ 0.7, safe pollution level
(ii) 0.7 < Ii ≤ 1.0, clean environment
(iii) 1.0 < Ii ≤ 2.0, slight pollution
(iv) 2.0 < Ii ≤ 3.0, moderate pollution
(v) Ii ≥ 3.0, serious/very high pollution

### Statistical analysis

The non-parametric Kruskal–Wallis test was applied for PTE contents in soil samples, as the data did not show a normal distribution using Shapiro–Wilk W normality or Levene variance tests. Spearman’s ranked correlation was used to determine the relationships among soil chemical properties, total element contents in soil, and bioaccessible contents of these elements. Statistical evaluation of the entire dataset was performed using STATISTICA 13.3 software (www.statsoft.com).

## Results

### Soil chemical parameters

Table 1 shows the chemical characteristics (mean ± SD) of the soils at the study site; the raw data are presented in Supplementary Tables S1 and S2. The soil reaction was slightly acidic (pH 5.9–6.5) with a CEC of 133–155 mmol L^−1^ at all the locations, providing a steady supply of nutrients to plants. The total C (3.5–4.0%) and N (0.27–0.30%) described the evident condition of soils in the temperate zone and supported the relative fertility of the soil. The C/N ratio was approximately 13 in all locations, supporting the mineralization of PTEs and other elements (Table 2). The total content of the PTE in soils at LKA, LKB, and LKC ranged from 40 to 65 mg kg^−1^ for Cd, from 3183 to 3896 mg kg^−1^ for Pb, and from 5108 to 6553 mg kg^−1^ for Zn and did not differ significantly (*p* < 0.05) among the sampling sites. The contents of Cd, Pb, and Zn were above the permissible limits, according to WHO and Czech Public Notice No. (153/2016). The mobile proportions of elements are summarised in Table 2. In contrast to the total element contents, all element contents differed significantly (*p* < 0.05) among the individual sampling sites. According to Czech Public Notice No. (153/2016), the mobile element contents exceeded the values indicative of a potential risk for crop contamination as well as for plant growth and soil biological values (i.e. 0.04 mg kg^−1^ Cd, 1.5 mg kg^−1^ Pb, 20 mg kg^−1^ Zn). However, compared to the total element contents in the soils, the mobile proportions were relatively low, representing 3.8–6.9% of Cd, 0.05–0.1% of Pb, and 2.5–5.1% of Zn of the total element content.

**Table 1:** Physicochemical characteristics (mean ± SD) of soils in the site. The Kruskal–Wallis test detected the *p*-value

Site	*n*	pH [0.1MCaCl_2_]	CEC [mmol L^−1^]	Total N [%]	Total C [%]
LKA	3	6.2 ± 0.02	133 ± 2.8	0.27 ± 0.01	3.5 ± 0.02
LKB	3	6.5 ± 0.03	155 ± 2.8	0.30 ± 0.01	4.0 ± 0.2
LKC	3	5.9 ± 0.02	135 ± 1.4	0.30 ± 0.01	4.0 ± 0.02
Mean		6.2 ± 0.3	141 ± 12	0.29 ± 0.01	3.8 ± 0.02
*p*-value		0.367	0.156	0.246	0.180

*LKA* Litavka location A, *LKB* Litavka location B, and *LKC* Litavka location C; *n* number of samples

**Table 2:** Total and extractable with 1M NH_4_NO_3_ (mean ± SD) contents of elements in soils in the site. The Kruskal–Wallis test detected the *p*-value

Site	*n*	Cd [mg kg^−1^]		Pb [mg kg^−1^]		Zn [mg kg^−1^]	
		Total	Extractable	Total	Extractable	Total	Extractable
LKA	3	40 ± 2	2.73 ± 0.03	3183 ± 218	2.14 ± 0.11	5109 ± 160	217 ± 4
LKB	3	65 ± 11	2.47 ± 0.09	3897 ± 392	1.84 ± 0.05	6553 ± 345	163 ± 2
LKC	3	54 ± 10	3.71 ± 0.10	3521 ± 330	3.53 ± 0.08	6302 ± 598	321 ± 9
Mean		53 ± 13	2.97 ± 0.57	3536 ± 416	2.51 ± 0.78	5988 ± 757	234 ± 70
*p*-value		0.051	0.027	0.099	0.027	0.061	0.043

*LKA* Litavka location A, *LKB* Litavka location B, and *LKC* Litavka location C; *n* number of samples

### Contamination level and correlation among soil parameters and mobile element proportions

The contamination levels of soils estimated as individual pollution indices in the 3 locations are given in Table 3, confirming the extremely high pollution level of all investigated areas. The organic matter component of the soil indicated by the C and N contents confirmed the close relationships of these soil parameters (Table 4) and, expectably, the significant (*p* < 0.05) relationships with the CEC levels. The closest significant (*p* < 0.05) correlations were recorded for the interrelationships of the mobile proportions of elements in soils. Among the soil properties, the mobile proportions were strongly negatively related to both the soil pH and CEC.

**Table 3:** The individual pollution indices (I_i_) of soils from the three locations

	Czech Public Notice No. 153/2016 (mg kg^−1^)	I_i_		
		LKA	LKB	LKC
Cd	0.5	80	130	106
Pb	60	53	65	109
Zn	50	102	70	126

*LKA* Litavka location A, *LKB* Litavka location B, and *LKC* Litavka location C

**Table 4:** Spearman rank correlation coefficients (*ρ*) among chemical properties of soils and mobile (1M NH_4_NO_3_ extractable) contents of risk elements

Variable	N [%]	C [%]	CEC [mmol L^−1^]	pH_CaCl2_	Cd mg kg^−1^	Pb mg kg^−1^	Zn mg kg^−1^
N		0.971*	0.618*	0.212	0.118	− 0.088	0.118
C			0.714*	0.089	0.029	− 0.086	0.029
CEC				0.600	− 0.657*	− 0.591	− 0.657*
pH					− 0.886*	− 1.000*	− 0.886*
Cd						0.886*	1.000*
Pb							0.886*
Zn							

The asterisks (*) indicate significant correlation at *p* < 0.05

### Herbaceous plants and PTE accumulation potential

**Fig. 2:**
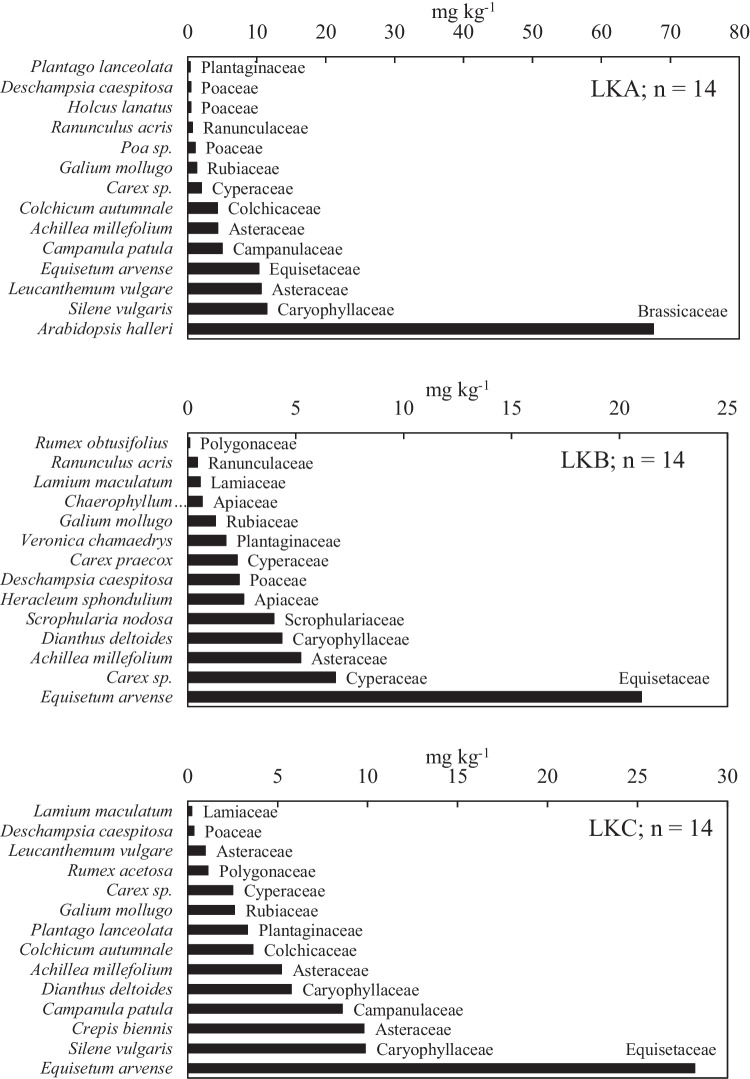
The total content of Cd in shoots of individual herbaceous plant species and their corresponding botanical families; *n* number of plant species

**Fig. 3:**
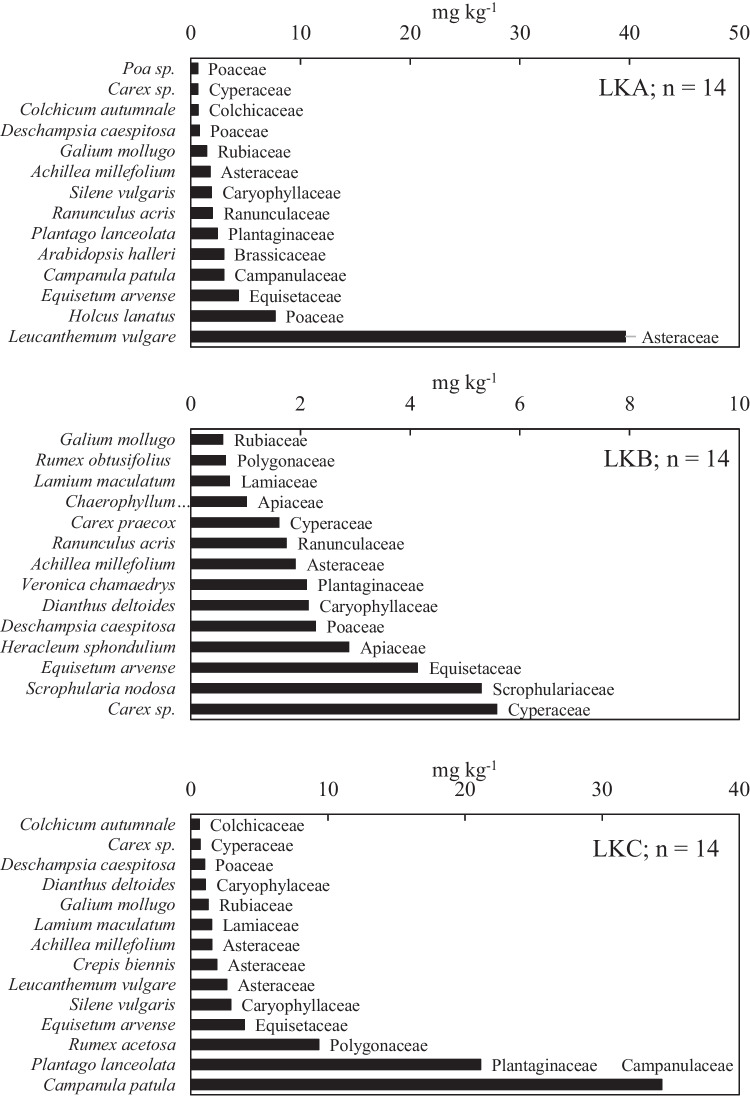
The total content of Pb in shoots of individual herbaceous plant species and their corresponding botanical families; *n* number of plant species

**Fig. 4:**
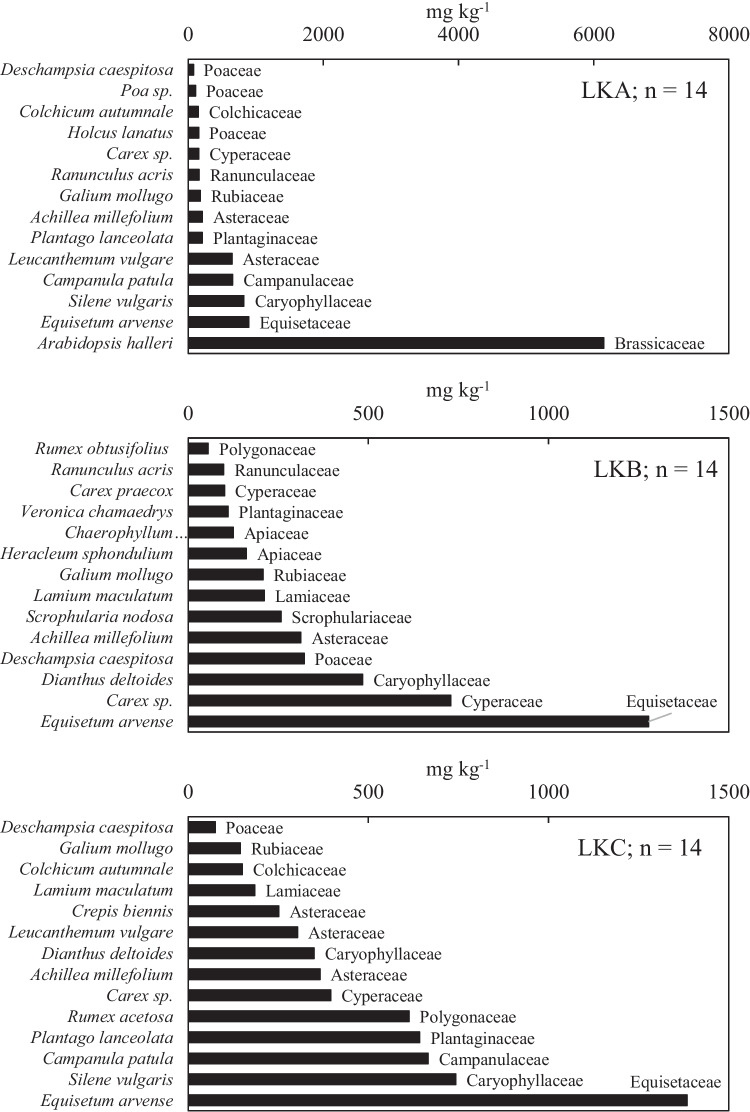
The total content of Zn in shoots of individual herbaceous plant species and their corresponding botanical families; *n* no of plant species

There were 15, 13, and 12 herbaceous plants identified in LKA, LKB, and LKC, respectively. In total, 23 perennial herbaceous species (16 dicots, 6 monocots, and 1 fern) and 15 botanical families were recorded due to species overlap in location. The Cd, Pb, and Zn contents in the studied biomass are shown in Figs. 2, 3, and 4, the raw data are presented in the Supplementary Table S3. Herbaceous species had a substantial effect on the Cd, Pb, and Zn content per locality.

The number of species found within the individual families was different. Nine families (Brassicaceae, Campanulaceae, Colchicaceae, Equisetaceae, Lamiaceae, Ranunculaceae, Rubiaceae, and Scrophulariaceae) were represented by only one species; four families (Apiaceae, Caryophyllaceae, Cyperaceae, Plantaginaceae, and Polygonaceae) were represented by two species; and two families (Asteraceae and Poaceae) were represented by three species (Table 5).

**Table 5:** Number of species forming botanical families

Family	*Species*	*N*. of species
Asteraceae	*Leucantamum vulgare*, *Achillea millifollium*, *Crepis biennis*	3
Apiaceae	*Chaerophyllum aromaticum*, *Heracleum spondulium*	2
Brassicaceae	*Arabidopsis halleri*	1
Cyperaceae	*Carex spp*., *Carex praecox*	2
Campanulaceae	*Campanula patula*	1
Colchicaceae	*Colchicum autumunale*	1
Caryophyllaceae	*Silene vulgaris*, *Dianthus deltoides*	2
Equisetaceae	*Equisetum arvense*	1
Lamiaceae	*Lamium maculatum*	1
Poaceae	*Deschampsia caespitosa*, *Holcus lanatus*, *Pos sp.*	3
Polygonaceae	*Rumex obtusifilius, Rumex acetosa*	2
Rubiaceae	*Galium mollugo*	1
Plantaginaceae	*Plantago lanceolata*, *Veronica chamaedrys*	2
Ranunculaceae	*Ranunculus acris*	1
Scrophulariaceae	*Scrophularia nodosa*	1

The Cd content was 0.4 mg kg^−1^ in *P*. *lanceolata*, and the highest accumulation of 68 mg kg^−1^ was observed in *Arabidopsis halle*ri in LKA (Fig. 2a). In location LKB, 0.1 mg kg^−1^ Cd was found in *Rumex obtusifolius*, and up to 21 mg kg^−1^ was found in *Equisetum arvense*. In LKC, there was 0.2 mg kg^−1^ in *Lamium maculatum* and 28.2 mg kg^−1^ in *E*. *arvense* (Fig. 2b and c). *Arabidopsis halleri* accounted for the only species in Brassicaceae, but it had a higher Cd and Zn (6151 mg kg^−1^) accumulation than all other species (Fig. 2a).

Furthermore, the Pb content in LKA was 0.63 mg kg^−1^ in *Poa* spp. and 40 mg kg^−1^ in *Leucanthemum vulgare* L. (Fig. 3a). The shoots of all the plants in LKB contained less than the published average upper limits in plants (10 mg Pb kg^−1^ (Kabata-Pendias 2011). The Pb content ranged from 0.60 mg kg^−1^ in *Gallium mollugo* to 5.6 mg kg^−1^ in *Carex* spp. in LKB and from 0.60 mg kg^−1^ in *Colchicum autumnale* to 34.3 mg kg^−1^ in *Campanula patula* in LKC (Fig. 3b and c). Moreover, *L*. *vulgare* was the only species that recorded exactly the value in plants (fodder for animals), according to the Czech Republic limit.

The Zn content ranged from 75 mg kg^−1^ in *D*. *caespitosa*. to 6151 mg kg^−1^ in *A*. *halleri* at location LKA (Fig. 4a), from 56 mg kg^−1^ in *R*. *obtusifolius* to 1277 mg kg^−1^ in *E*. *arvense* at LKB (Fig. 4b), and from 75 mg kg^−1^ in *D*. *caespitosa* to 1384 mg kg^−1^ in *E*. *arvense* at LKC (Fig. 4c).

### Bioaccumulation factor-BAF_shoot_

Except for *A*. *halleri*, BAF_shoot_ values for Cd and Zn for all the studied herbaceous plants were less than 1 (Table 6). Additionally, *A*. *halleri* had a high Cd and Zn content (BAF_shoot_ > 1). The BAF_shoot_ for Pb in all plants was < 1.

**Table 6:** Bioaccumulation factor (BAF) of studied plant species for each element in all the locations

Herbaceous species	Cd			Pb			Zn		
	LKA	LKB	LKC	LKA	LKB	LKC	LKA	LKB	LKC
*Plantago lanceolata*	< 0.1	-	0.06	< 0.1		< 0.1	< 0.01	-	< 0.1
*Deschampsia caespitosa*	< 0.1	< 0.1	< 0.1	< 0.1	< 0.1	< 0.1	< 0.01	< 0.1	< 0.1
*Holcus lanatus*	< 0.1	-	-	< 0.1		-	< 0.01	-	-
*Ranunculus acris*	< 0.1	< 0.1	-	< 0.1	< 0.1	-	< 0.01	< 0.1	-
*Chaerophyllum aromaticum*	< 0.1	-	-	< 0.1	-	-	< 0.01	-	-
*Poa sp*	< 0.1	-	-	< 0.1	-	-	< 0.01	-	-
*Galium mollugo*	< 0.1	< 0.1	0.05	< 0.1	< 0.1	< 0.1	< 0.01	< 0.1	< 0.1
*Carex sp*	< 0.1	< 0.1	0.05	< 0.1	< 0.1	< 0.1	< 0.01	< 0.1	0.06
*Colchicum autumnale*	0.11	-	0.07	< 0.1	-	< 0.1	< 0.01	-	-
*Campanula patula*	0.13	-	0.16	< 0.1	-	< 0.1	0.13	-	-
*Achillea millefolium*	0.13	0.08	-	< 0.1	< 0.1	-	0.01	< 0.1	< 0.1
*Equisetum arvense*	0.26	0.32	0.52	< 0.1	< 0.1	< 0.1	0.18	0.19	0.22
*Leucanthemum vulgare*	0.28	-	< 0.1	< 0.1		< 0.1	0.13	-	-
*Silene vulgaris*	0.29	0.15	-	< 0.1	< 0.1	-	0.16	0.11	-
***Arabidopsis halleri***	1.7*	**-**	**-**	< 0.1	**-**	**-**	1.2*	-	< 0.1
*Dianthus deltoides*	-	0.07	0.11	-	< 0.1	< 0.1	-	0.07	< 0.1
*Scrophularia nodosa*	*-*	0.06	-	-	< 0.1	-	-	< 0.1	< 0.1
*Carex praecox*	*-*	< 0.1	-	-	< 0.1	-	-	< 0.1	< 0.1
*Veronica chamaedrys*	*-*	< 0.1	-	-	< 0.1	-	-	< 0.1	< 0.1
*Lamium maculatum*	*-*	< 0.1	< 0.1	-	< 0.1	< 0.1	-	< 0.1	< 0.1
*Rumex obstusifolius*	*-*	< 0.1		-	< 0.1	-	-	< 0.1	0.1
*Crepis biennis*	*-*	-	0.18	-	-	< 0.1	-	-	-
*Rumex acetosa*	-	-	< 0.1	-	-	< 0.1	-	-	-

Bolded values with asterisks (*) indicate a high accumulation of the risk element; *LKA* Litavka location A, *LKB* Litavka location B, and *LKC* Litavka location C

## Discussion

This exploratory study revealed Cd, Pb, and Zn accumulation by different herbaceous species at a highly contaminated site in Příbram, Czech Republic. Evidence of PTE accumulation by plants is supported by the comparative differences in the Cd, Pb, and Zn contents. A soil geochemical exploration by Borůvka and Vácha (2006) showed maximum *Aqua regia* contents of 129, 8445, and 11739 mg kg^−1^ for Cd, Pb, and Zn, respectively. The soils in this study were still contaminated with multiple PTEs, especially Cd, Pb, and Zn, as confirmed by the extremely high individual pollution indices. Although the mobile proportions of elements were relatively low, there were adverse effects of these element contents on crop production, plant growth, and soil biological values. Similarly, the impact of mining activities on risk element (Pb, As) uptake by various vegetables was evaluated by Salas‑Muñoz et al. (2022), who showed that the individual pollution indices classified the soil as polluted. Subsequently, the element levels in the various vegetable species exceeded the national limits for the maximum allowable levels of these elements in vegetables, where the highest levels of Cd were found in pepper (*Capsicum annum*) fruit. Therefore, such locations should be considered unsuitable for crop production. As a result of pollution, most of the herbaceous species studied accumulated excess Cd, Pb, and Zn above their allowed limits. However, plant species with excess accumulation of Cd, Pb, and Zn in their shoots can be considered for the remediation of soils contaminated with PTE.

### Soil characteristics and risk element mobility, and their implications

The studied PTE contents in the soils of this study threaten human health, and the accumulation in plant tissue (shoot) makes the site unsuitable for crop production. The relatively high CEC is a useful indicator of soil fertility because soil can still supply cationic elements (Ca^2+^, K^2+^, and Mg^2+^) to grow herbaceous species even though the soil has excess PTE (Ćirić et al. 2023; FAO 2024). The CEC in this study is comparatively similar to other PTE-contaminated fields in the study area at which herbaceous species grow (Asare et al. 2023a, 2024). The negative correlation of CEC levels and mobile proportions of PTEs confirms the positive role of the soil sorption complex in the potential reduction of element bioaccessibility in the soil. However, the weak correlation between CEC and the components of organic matter (C and N) indicates that the soil hardly binds to cations (e.g. Cd^2+^ and Zn^2+^) and can contribute to the uptake of free ions by plants. According to the negative relationship between pH and C and N, the reduction in adsorbed cations suggests the mobility of free ions (Zhou et al. 2019; Solly et al. 2020). The ability of the soil to hold elements was shown in the values of the C/N ratio, which indicates nutrient mineralization and the PTE content. Moreover, most crops do best with mineral soils of pH 6.5, but in this state, PTEs mineralise and may cause bioaccumulation by both fauna and flora (FAO 2024; Asare et al. 2024). The mobile proportions of PTEs showed a strong negative correlation with soil pH, confirming the determining position of soil pH in the bioaccessibility of the cationic PTEs. The C and N contents, forming the organic matter component of the soil, are relatively high and similar to soils with even the application of compost and sewage sludge within the same vicinity as a short coppice rotation plantation with *Salix* and *Populus* (Asare et al. 2024).

### PTE accumulation in plant shoots

The accumulation of PTEs in plants growing in soils polluted by mining activity can result in the adverse effects of these elements on plant physiology and biochemistry. For instance, phytotoxicity symptoms, such as necrosis of the aerial parts and reduction of plant width, fresh aerial biomass, and leaf area, were reported in *Lactuca sativa* plants by Calabró et al. (2022); the plants were planted in soil amended by polluted mine waste. The Cu, Pb, and Zn bioaccumulation was affected by the bioaccessibility of the elements in the mine waste and reached up to 1453 ± 220 mg kg^−1^ of Zn, 277 ± 18 mg kg^−1^ of Pb, and 2553 ± 25 mg kg^−1^ of Cu. Certain plant species accumulate PTEs in aboveground organs (stem, appendages, leaves, lateral buds, flowering stems, and flower buds), with minimal or no signs of toxicity (Memon 2016; Suman et al. 2018; Ashraf et al. 2019). Aboveground accumulators usually sequester PTEs in their leaf cells, form chelates in the cell walls, and encourage detoxification, allowing them to accumulate high contents (Skuza et al. 2022; Asare et al. 2023b). The phytoextraction of PTEs in the aboveground organs of plants prevents surface soils from contaminating other areas via runoff and erosion (Yan et al. 2020).

The Cd and Zn content in all herbaceous species represented an increase of 1.2 and > 90 times, respectively, compared to the WHO limit, except for *R*. *obtusifolius* (Table 7). Meanwhile, *A*. *halleri* and *Equisetum arvense* exhibited the highest tendencies for appropriate Cd remediation in the soils for all studied locations studied. *Arabidopsis halleri* is the most suitable candidate plant for phytoremediation of Cd-contaminated soil. Among all investigated plants, only this species fulfilled the criteria of hyperaccumulating plants as stated by van der Ent et al. (2013). Studies have shown that *A*. *halleri* promotes Cd solubilization of Cd in the soil directly or indirectly using its secreted root exudates (Ariyanti et al. 2023). Moreover, *A*. *halleri*, a member of the Brassicaceae family, is well known for its ability to hyperaccumulate Zn and Cd from contaminated soils (Grignet et al. 2022). Tlustoš et al. (2016) showed the phytoremediation potential of *A*. *halleri* planted under field conditions in the vicinity of the sampling area of this study and found PTE removal per season of 0.02–0.17% for Cd and 0.004–0.20% for Zn. The low phytoremediation efficiency is due to low annual biomass production not exceeding 1.5 tonnes per hectare under field conditions. The current study also showed the suitability of *Equisetum arvense* in removing Zn from the soil, which was evident based on its ability to accumulate the highest contents of this element. Except for *L*. *maculatum*, the Cd content in all studied species was above the published average upper limits in plants, i.e. 0.2 mg kg^−1^ (Kabata-Pendias 2011).

**Table 7:** Regulatory limits of studied potentially toxic elements in the shoot of herbaceous plants

PTE	Limit			
	Czech Republic decree [mg kg^−1^]	WHO (for plants) [mg kg^−1^]	^a^Kabata-Pendias (2011) [mg kg^−1^]	^b^Souza et al. (2020) [mg kg^−1^]
Cd	0.2	0.2	0.2	
Pb	40	2.0	10	
Zn		0.6	150	> 300

Czech Republic decree: Commission Directive 2010/6/EU, WHO 1996, ^a^according to Kabata-Pendias (2011), ^b^according to Souza et al. (2020)

Although the Pb content according to WHO was above the permissible limits in 10 species (*L*. *vulgare*, *E*. *arvense*, *Rumex acetosa*, *P*. *lanceolata*, *C*. *patula*, *Veronica chamaedrys*, *Dianthus deltoides*, *D*. *caespitosa*, *S*. *vulgaris*, *Scrophularia nodosa*, and *Carex* sp.), only *L*. *vulgare*, *C*. *patula*, *P*. *lanceolata*, *E*. *arvense*, and *Rumex acetosa* were above the published average upper limits in plants, i.e. 10 mg Pb kg^−1^ (Kabata-Pendias 2011). Therefore, soil–root–shoot transport and the shoot accumulation ability of Pb should be assessed in more detail at the investigated location. However, Pb uptake by plants can be affected by the low bioaccessibility of Pb in the research area.

The accumulation statuses of the shoots of these plants suggest their suitability for the phytoextraction of varied levels of Cd-, Pb-, and Zn-contaminated soils. Plants with the highest accumulation are the most suitable for cleaning contaminated soils. However, the regulatory limits are used for benchmarks to avoid ecotoxicological incidences, e.g. FAO/WHO. For the effective phytoextraction of risk elements from the polluted soils, not only is the high bioaccumulation ability of plants necessary but also the high production of biomass per annum, resulting in substantial risk element removal from the polluted soil. For instance, the Zn and Pb phytoextraction potential of *Helianthus annuus* was highlighted by Aybar et al. (2023). Similarly, Aladesanmi et al. (2019) reported the promising ability to bioaccumulate Cd, Cr, Pb, Cu, and Zn by *Zea mays* grown on soils amended by various mining and smelting wastes as well as wastes from industrial activities. Therefore, the choice of plants with high biomass production and good PTE accumulation ability can be more effective than hyperaccumulating plants, such as *A*. *halleri*.

PTE accumulation is species specific, with less evidence to generalise accumulation according to family, as plants belonging to similar botanical families show varying accumulation patterns. For example, *Plantago lanceolata* and *V*. *chamaedrys* belong to the Plantaginaceae family, but the former recorded 21 mg Pb kg^−1^ (in LKC) from the total accumulation of 26 mg kg^−1^. Individual species of herbaceous plants from the Brassicaceae and Equisetaceae families accumulated Cd and Zn even higher than families represented by more than one species, e.g. Asteraceae and Poaceae. Furthermore, Asteraceae represents the family with the highest Pb accumulation, but individual species, C. *biennis* and *A*. *millefolium*, recorded lower contents than *L*. *vulgare* (40 mg kg^−1^). Plant species in the Equisetaceae family obtained the highest Cd accumulation in LKB and LKC, with the overall highest recorded by Brassicaceae in LKA. Comparing the Zn content in the soil with the levels of accumulation by Asteraceae, Brassicaceae, Equisetaceae, Campanulaceae, Caryophyllaceae, Cyperaceae, Plantaginaceae, and Polygonaceae, representing families with more than 300 mg of Zn kg^−1^ (of which most plants show Zn phytotoxicity in their dry weight; Long et al. 2003), these plants should be considered in potential phytoremediation measures at the investigated area.

In addition to *A*. *halleri*, *E*. *arvense* showed promising Cd accumulation potential in its shoots. Therefore, a more detailed study is necessary to assess the soil Cd removal ability of this plant. *Equisetum arvense* persists in highly contaminated sites (Dradrach et al. 2020). As a medicinal herb, *E*. *arvense* produces isoquercitroside from the aerial part and exhibits therapeutic potential, but requires critical analysis before use (European Medicine Agency 2016; Boeing et al. 2021), especially of those grown at contaminated sites. Herbaceous species, such as *A*. *millefolium*, *P*. *lanceolata*, *A*. *arvense*, and *E*. *vulgare*, are vital medicinal plants characterised by their increased ability to accumulate PTEs, which requires strict awareness of regulatory limits before use (European Medicine Agency 2016). Most species accumulated high Cd levels above this limit. It is pertinent to compare the content of these PTEs to regulatory limits according to the WHO, especially for medicinal herbaceous species, e.g. *Matricaria recutita* and other plants with human consumable metabolites. Moreover, most of the studied herbaceous plants are livestock resources.

The herbaceous plants studied, except for *L*. *vulgare*, showed lower Pb accumulation in the shoot compared to the safe limit for animal feed, following Czech Republic regulations (Commission Directive 2010/6/EU). Many plants exhibit restricted Pb translocation in aerial parts due to blockage by Casparian bands in the roots, while others reduce the mobility and uptake of PTEs through root fixation, e.g. *Trifolium repens* and *Rumex alpinus* (Bidar et al. 2008; Lambrechts et al. 2014; Jungová et al. 2022). Many species exhibit a high Pb content in the roots due to selective translocation (e.g. Bidar et al. 2008; Marques et al. 2009).

Zinc toxicity signs are usually > 300 mg kg^−1^ in the leaf, while some plants, including *Mimosa caesalpiniaefolia*, are visible at content < 100 mg Zn kg^−1^ (Souza et al. 2020). *Rumex acetosa*, *C*. *patula*, *S*. *vulgaris*, and *E*. *arvense* had high accumulation of this element (> 300 mg Zn kg^−1^). Six species from LKA and LKB, together with seven from LKC, accumulated > 300 mg Zn kg^−1^, with* E*. *arvense* having more than 1000 mg kg^−1^ in some cases. Additionally, *Equisetum* species, e.g. *E*. *hyemale*, possess deep root systems that can extract a higher Pb and Cd content than other plants (Kurniati et al. 2014).

Although the soil conditions were relatively similar in all locations, plants showed different patterns of shoot accumulation, resulting from their varied genotypes, which may have different uptake and accumulation mechanisms. In contrast, some plants are susceptible to specific PTEs (Asare et al. 2023b, a). Studies have shown that different plants exhibit varied phytoremediation responses. For example, *Lotus corniculatus* and *T*. *repens* of the Fabaceae family make a significant contribution to phytostabilisation (Bidar et al. 2008; Amer et al. 2013), and *Medicago lupulina* contributes to Pb accumulation in its aerial parts (Amer et al. 2013). Moreover, different plants produce exudates and select microbes in the rhizosphere, supporting PTE dissolution and promoting effective uptake (Bais et al. 2006; Sun et al. 2020). The roots of many plants have adaptations and attractants for some enzymes capable of reworking PTEs for possible uptake (Gianfreda 2015).

## Conclusions

This exploratory study revealed the accumulation capacity of the shoots of herbaceous plant species to Cd, Pb, and Zn from contaminated soils. Differences in their accumulation are vital in identifying appropriate species for potential phytoremediation use. PTE accumulation by herbaceous plants above the permissible limit indicates that the food chain can be affected if such fields are used for crop production. Except for *Rumex obtusifolius*, all herbaceous species studied had shoot accumulation of Cd above the permissible limit of the WHO. The Zn content in all plants was higher than the allowable limit, and 10 species were higher than the limit for Pb. Moreover, *Arabidopsis halleri* and *Equisetum arvense* accumulated Cd and Zn according to their relatively high contents compared to other plants. *Arabidopsis halleri* had a BAF > 1 for Cd and Zn, confirming its hyperaccumulation potential. *Equisetum arvense* showed promising accumulation ability of these elements, and more detailed investigations can be recommended to elucidate the potential phytoremediation ability of this plant species. Herbaceous plants with PTE accumulations higher than threshold levels and characterised by high annual biomass production can be considered for the potential establishment of phytoremediation measures. However, the transport of risk elements in the soil–root–shoot system of these plant species and the total annual off-take of elements from the soil should be elucidated in detail in further research. Regardless of the low mobility of Pb in the soils, *Leucanthemum vulgare* exhibited relatively good Pb accumulation potential, with the highest shoot accumulation among all plant species, which was above the allowable limit for green feed.

## Supplementary Information

Below is the link to the electronic supplementary material.

Supplementary file1 (DOCX 26 KB)

## Data Availability

The data that support the findings of this study are available from the corresponding author upon reasonable request.
