# Supplementary material for: Abilities of herbaceous plant species to phytoextract Cd, Pb, and Zn from arable soils after poly-metallic mining and smelting
Source: Environ Sci Pollut Res Int. 2025 Mar 17;32(14):8834–49. doi: 10.1007/s11356-025-36241-6 (PMC11968566; doi:10.1007/s11356-025-36241-6)
Supplement: Supplementary file 1 — Supplementary file1 (DOCX 26 KB) [file 11356_2025_36241_MOESM1_ESM.docx]

**Supplementary material**

**Table S1**. The main characteristics of the experimental soils

| Site | N  [mg kg^-1^] | C  [mg kg^-1^] | CEC  [mg kg^-1^] | pH |
| --- | --- | --- | --- | --- |
| LKA | 0.285 | 3.68 | 135 | 6.26 |
|  | 0.250 | 3.26 | 131 | 6.22 |
|  | 0.268 | 3.47 | 133 | 6.24 |
| LKB | 0.285 | 3.81 | 153 | 6.27 |
|  | 0.312 | 4.28 | 157 | 6.74 |
|  | 0.299 | 4.04 | 155 | 6.51 |
| LKC | 0.304 | 4.01 | 136 | 5.87 |
|  | 0.304 | 3.95 | 134 | 5.91 |
|  | 0.304 | 3.98 | 135 | 5.89 |

**Table S2.** Total and extractable with 1M NH_4_NO_3_ contents of PTEs in the individual soil samples

| Site | Cd  [mg kg^-1^] | | Pb  [mg kg^-1^] | | Zn  [mg kg^-1^] | |
| --- | --- | --- | --- | --- | --- | --- |
|  | Total | Extractable | Total | Extractable | Total | Extractable |
| LKA | 39.4 | 2.70 | 3071 | 2.04 | 5059 | 213 |
|  | 37.5 | 2.76 | 3043 | 2.25 | 4973 | 221 |
|  | 42.2 | 2.73 | 3435 | 2.14 | 5294 | 217 |
| LKB | 76.4 | 2.39 | 4335 | 1.89 | 6849 | 161 |
|  | 62.4 | 2.56 | 3774 | 1.79 | 6638 | 164 |
|  | 55.9 | 2.47 | 3580 | 1.84 | 6173 | 163 |
| LKC | 44.5 | 3.60 | 3159 | 3.61 | 5644 | 312 |
|  | 53.7 | 3.81 | 3631 | 3.45 | 6447 | 330 |
|  | 63.7 | 3.71 | 3797 | 3.53 | 6815 | 321 |

**Table S3**. The complete dataset of the PTEs contents in the individual plant species

|  | **Species** | **Cd** | **Pb** | **Zn** |
| --- | --- | --- | --- | --- |
|  |  | **mg kg^-1^** | **mg kg^-1^** | **mg kg^-1^** |
| LKA | *Achillea millefolium* | 4.34 | 1.75 | 208 |
|  | *Silene vulgaris* | 11.5 | 1.87 | 825 |
|  | *Plantago lanceolata* | 0.350 | 2.41 | 210 |
|  | *Deschampsia caespitosa* | 0.424 | 0.777 | 76.5 |
|  | *Equisetum arvense* | 10.3 | 4.34 | 897 |
|  | *Holcus lanatus* | 0.431 | 7.67 | 155 |
|  | *Leucanthemum vulgare* | 10.6 | 39.6 | 650 |
|  | *Colchicum autumnale* | 4.30 | 0.653 | 148 |
|  | *Galium mollugo* | 1.25 | 1.43 | 180 |
|  | *Arabidopsis halleri* | 67.5 | 2.99 | 6151 |
|  | *Ranunculus acris* | 0.640 | 1.95 | 163 |
|  | *Campanula patula* | 4.96 | 3.01 | 658 |
|  | *Carex sp.* | 1.94 | 0.648 | 157 |
|  | *Poa sp.* | 1.06 | 0.625 | 109 |
| LKB | *Achillea millefolium* | 5.22 | 1.91 | 313 |
|  | *Snaerophyllum aromaticum* | 0.666 | 1.02 | 125 |
|  | *Heracleum sphondulium* | 2.57 | 2.88 | 161 |
|  | *Dianthus deltoides* | 4.36 | 2.15 | 484 |
|  | *Deschampsia caespitosa* | 2.37 | 2.28 | 322 |
|  | *Scrophularia nodosa* | 3.99 | 5.30 | 258 |
|  | *Equisetum arvense* | 21.0 | 4.14 | 1277 |
|  | *Carex sp.* | 6.83 | 5.58 | 729 |
|  | *Carex praecox* | 2.27 | 1.61 | 101 |
|  | *Ranunculus acris* | 0.441 | 1.74 | 98.0 |
|  | *Galium mollugo* | 1.27 | 0.590 | 208 |
|  | *Lamium maculatum* | 0.566 | 0.713 | 211 |
|  | *Rumex obtusifolius* | 0.074 | 0.639 | 55.6 |
|  | *Veronica chamaedrys* | 1.76 | 2.12 | 111 |
| LKC | *Achillea millefolium* | 5.22 | 1.52 | 366 |
|  | *Silene vulgaris* | 9.90 | 2.90 | 742 |
|  | *Deschampsia caespitosa* | 0.361 | 1.00 | 75.0 |
|  | *Crepis biennis* | 9.82 | 1.90 | 251 |
|  | *Dianthus deltoides* | 5.77 | 1.06 | 350 |
|  | *Leucanthemum vulgare* | 0.993 | 2.62 | 304 |
|  | *Plantago lanceolata* | 3.33 | 21.1 | 642 |
|  | *Equisetum arvense* | 28.2 | 3.91 | 1384 |
|  | *Campanula patula* | 8.61 | 34.3 | 666 |
|  | *Rumex acetosa* | 1.15 | 9.33 | 612 |
|  | *Carex sp.* | 2.53 | 0.677 | 395 |
|  | *Galium mollugo* | 2.63 | 1.27 | 145 |
|  | *Lamium maculatum* | 0.244 | 1.51 | 185 |
|  | *Colchicum autumnale* | 3.65 | 0.623 | 150 |
